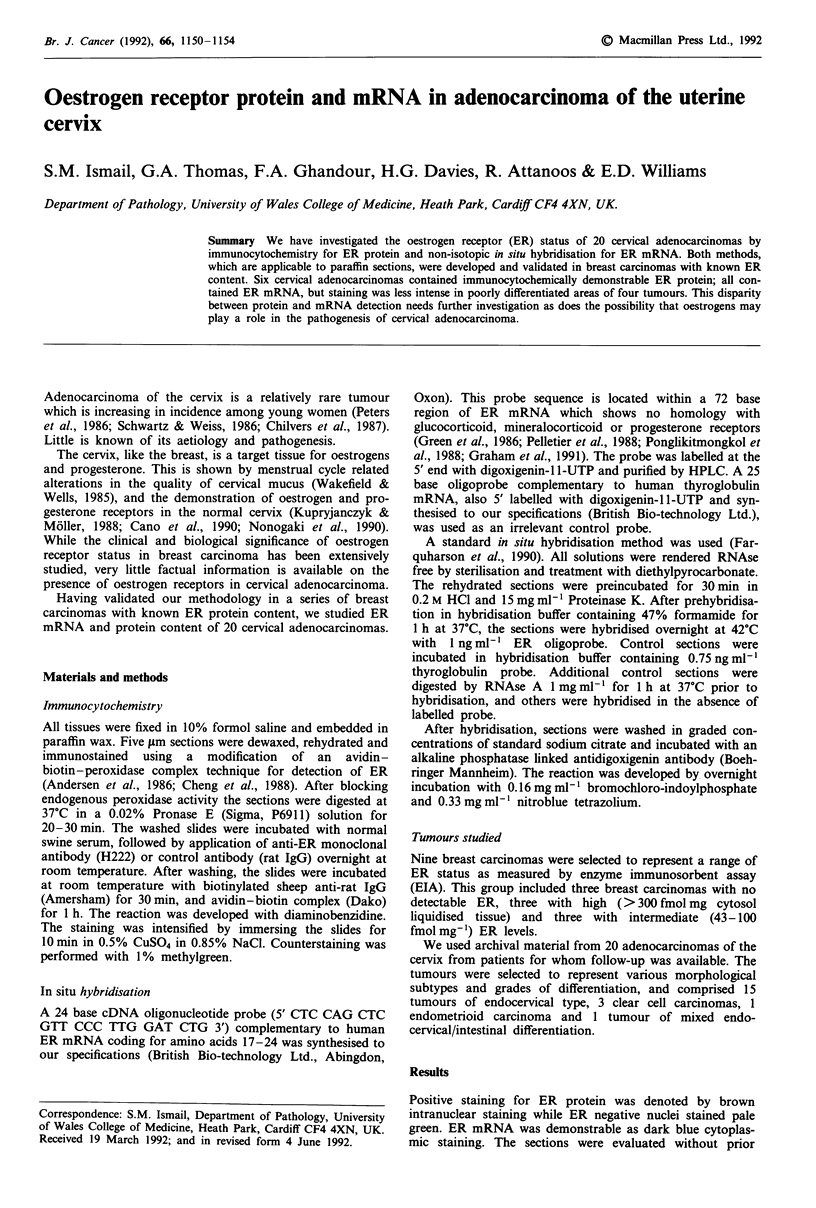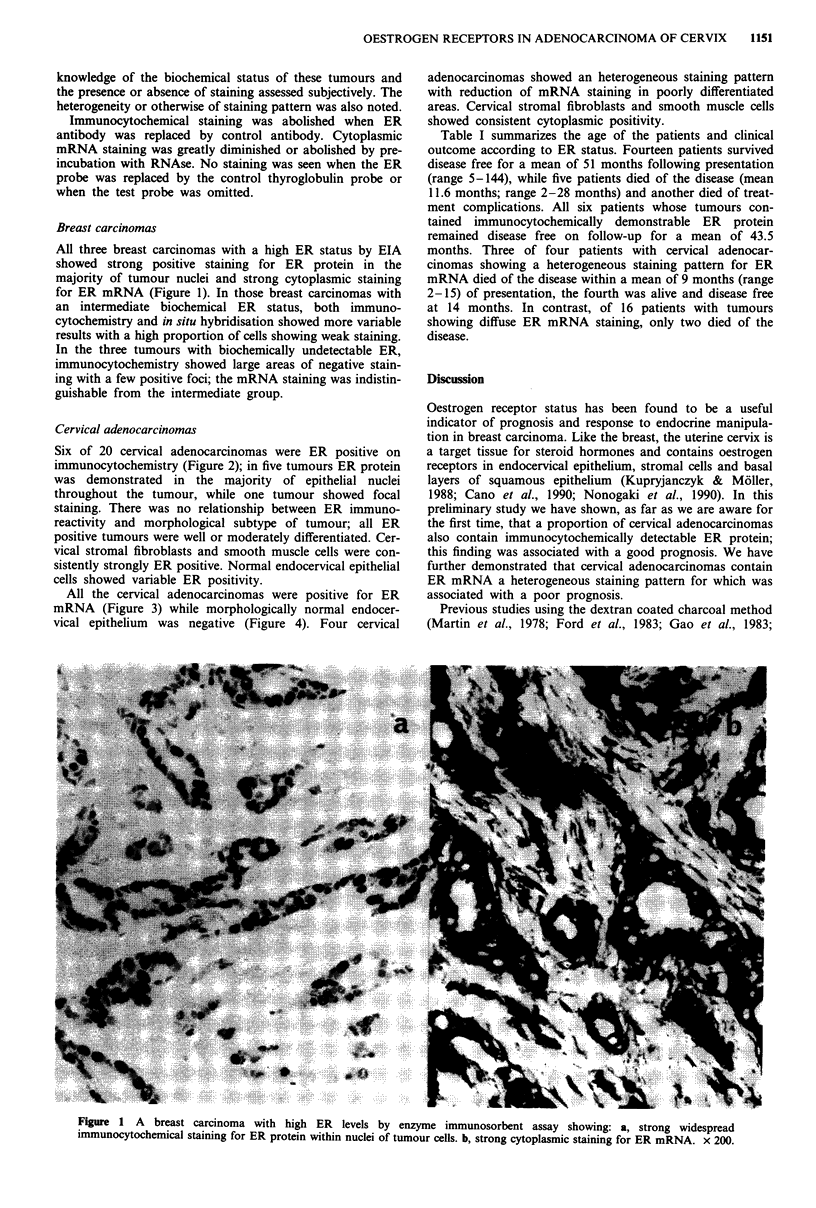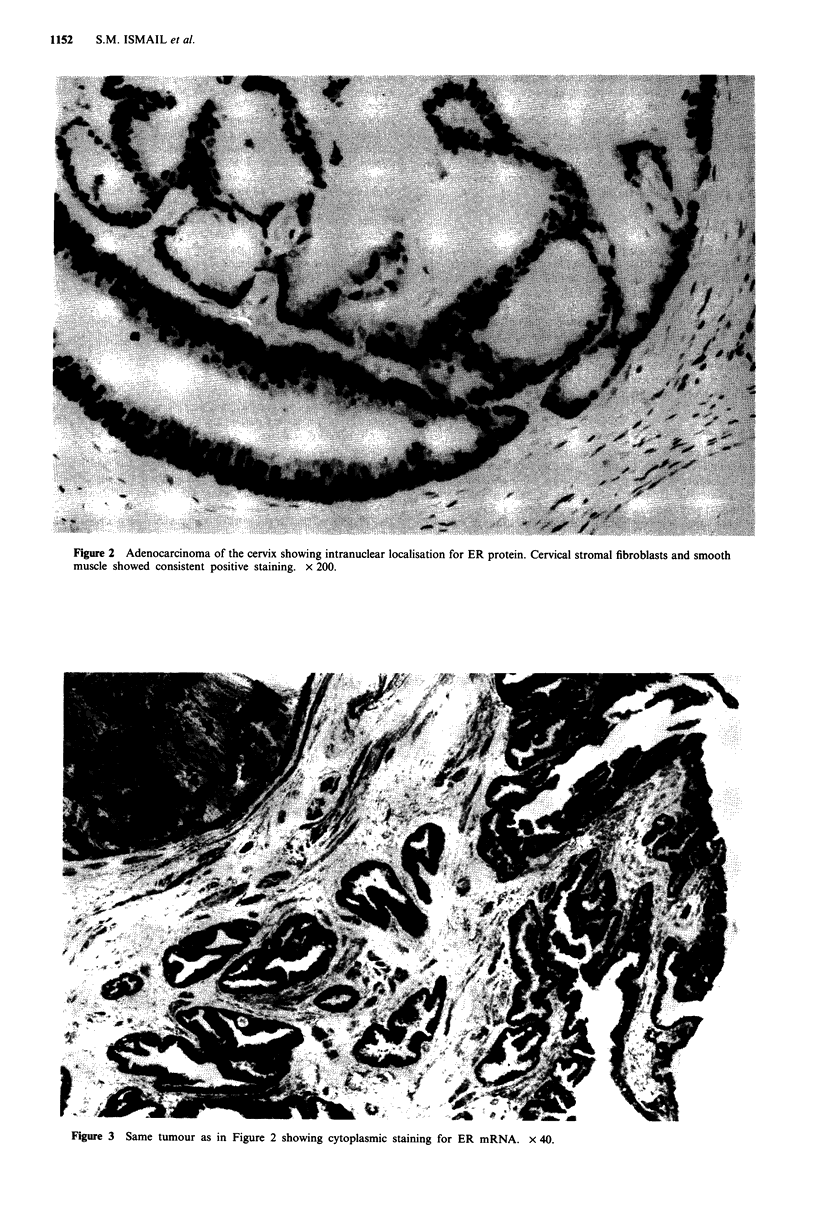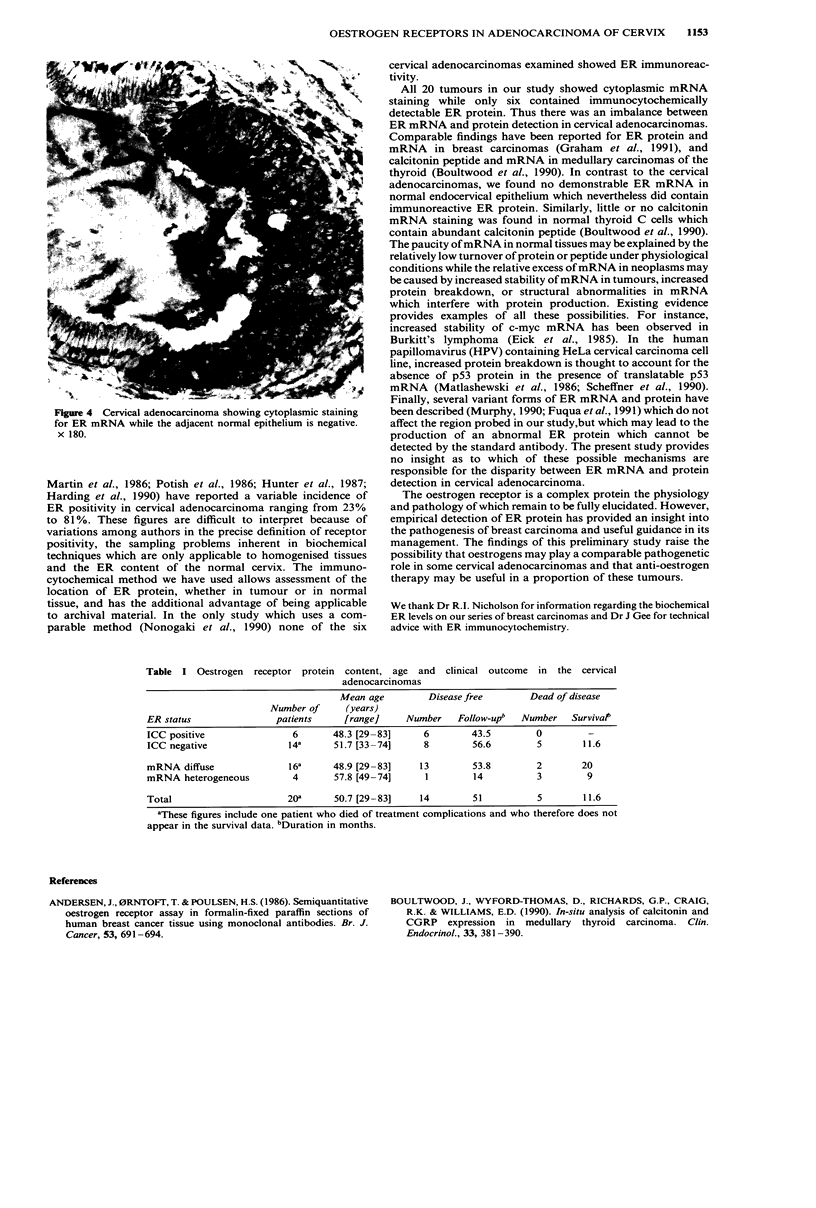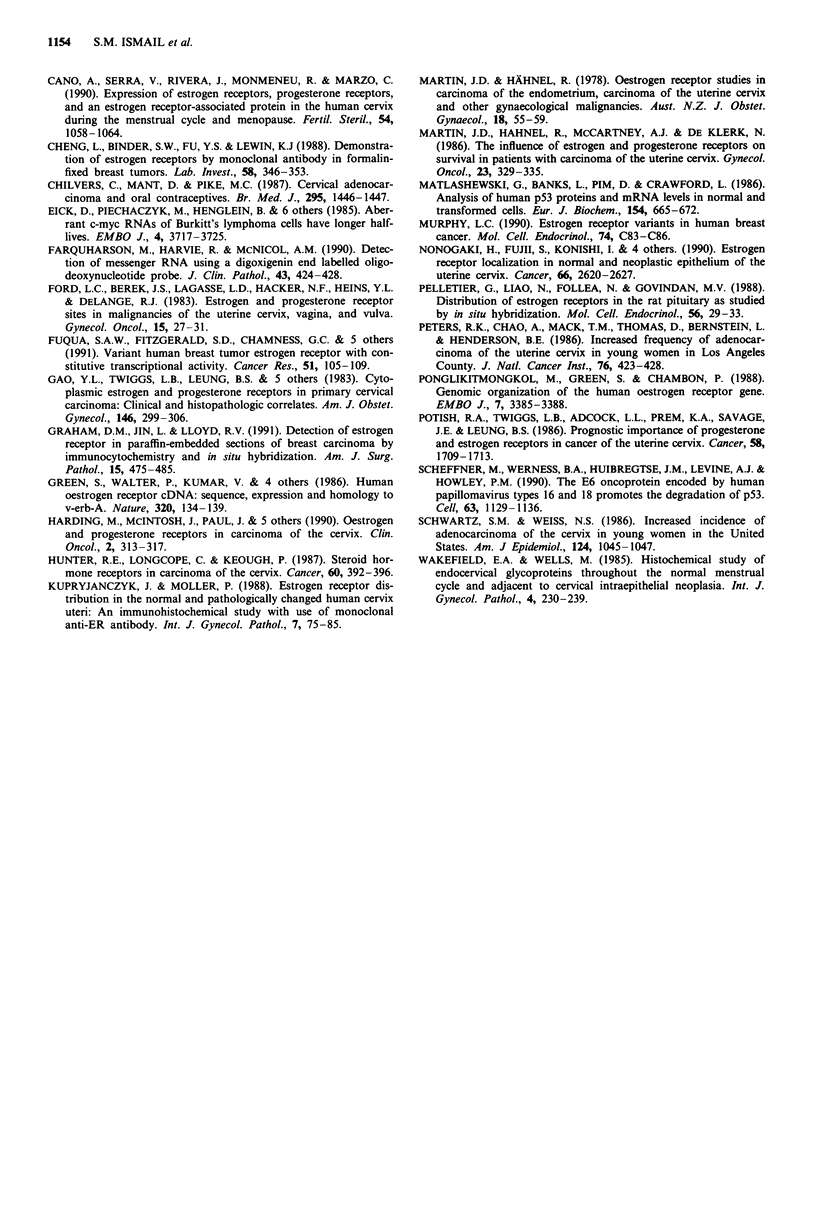# Oestrogen receptor protein and mRNA in adenocarcinoma of the uterine cervix.

**DOI:** 10.1038/bjc.1992.425

**Published:** 1992-12

**Authors:** S. M. Ismail, G. A. Thomas, F. A. Ghandour, H. G. Davies, R. Attanoos, E. D. Williams

**Affiliations:** Department of Pathology, University of Wales College of Medicine, Heath Park, Cardiff, UK.

## Abstract

**Images:**


*Br. J. Cancer* (1992), **66**, 1150–1154 © Macmillan Press Ltd., 1992

### Oestrogen receptor protein and mRNA in adenocarcinoma of the uterine cervix

S.M. Ismail, G.A. Thomas, F.A. Ghandour, H.G. Davies, R. Attanoos & E.D. Williams

*Department of Pathology, University of Wales College of Medicine, Heath Park, Cardiff CF4 4XN, UK.*

**Summary** We have investigated the oestrogen receptor (ER) status of 20 cervical adenocarcinomas by immunocytochemistry for ER protein and non-isotopic *in situ* hybridisation for ER mRNA. Both methods, which are applicable to paraffin sections, were developed and validated in breast carcinomas with known ER content. Six cervical adenocarcinomas contained immunocytochemically demonstrable ER protein; all contained ER mRNA, but staining was less intense in poorly differentiated areas of four tumours. This disparity between protein and mRNA detection needs further investigation as does the possibility that oestrogens may play a role in the pathogenesis of cervical adenocarcinoma.

Adenocarcinoma of the cervix is a relatively rare tumour which is increasing in incidence among young women (Peters *et al*., 1986; Schwartz & Weiss, 1986; Chilvers *et al*., 1987). Little is known of its aetiology and pathogenesis.

The cervix, like the breast, is a target tissue for oestrogens and progesterone. This is shown by menstrual cycle related alterations in the quality of cervical mucus (Wakefield & Wells, 1985), and the demonstration of oestrogen and progesterone receptors in the normal cervix (Kupryjanczyk & Möller, 1988; Cano *et al*., 1990; Nonogaki *et al*., 1990). While the clinical and biological significance of oestrogen receptor status in breast carcinoma has been extensively studied, very little factual information is available on the presence of oestrogen receptors in cervical adenocarcinoma.

Having validated our methodology in a series of breast carcinomas with known ER protein content, we studied ER mRNA and protein content of 20 cervical adenocarcinomas.

### Materials and methods

### *Immunocytochemistry*

All tissues were fixed in 10% formol saline and embedded in paraffin wax. Five μm sections were dewaxed, rehydrated and immunostained using a modification of an avidin–biotin–peroxidase complex technique for detection of ER (Andersen *et al*., 1986; Cheng *et al*., 1988). After blocking endogenous peroxidase activity the sections were digested at 37°C in a 0.02% Pronase E (Sigma, P6911) solution for 20–30 min. The washed slides were incubated with normal swine serum, followed by application of anti-ER monoclonal antibody (H222) or control antibody (rat IgG) overnight at room temperature. After washing, the slides were incubated at room temperature with biotinylated sheep anti-rat IgG (Amersham) for 30 min, and avidin–biotin complex (Dako) for 1 h. The reaction was developed with diaminobenzidine. The staining was intensified by immersing the slides for 10 min in 0.5% $CuSO_4$ in 0.85% NaCl. Counterstaining was performed with 1% methylgreen.

### In situ *hybridisation*

A 24 base cDNA oligonucleotide probe (5′ CTC CAG CTC GTT CCC TTG GAT CTG 3′) complementary to human ER mRNA coding for amino acids 17–24 was synthesised to our specifications (British Bio-technology Ltd., Abingdon, Oxon). This probe sequence is located within a 72 base region of ER mRNA which shows no homology with glucocorticoid, mineralocorticoid or progesterone receptors (Green *et al*., 1986; Pelletier *et al*., 1988; Ponglikitmongkol *et al*., 1988; Graham *et al*., 1991). The probe was labelled at the 5′ end with digoxigenin-11-UTP and purified by HPLC. A 25 base oligoprobe complementary to human thyroglobulin mRNA, also 5′ labelled with digoxigenin-11-UTP and synthesised to our specifications (British Bio-technology Ltd.), was used as an irrelevant control probe.

A standard *in situ* hybridisation method was used (Farquharson *et al*., 1990). All solutions were rendered RNAse free by sterilisation and treatment with diethylpyrocarbonate. The rehydrated sections were preincubated for 30 min in 0.2 M HCl and 15 mg $ml^{-1}$ Proteinase K. After prehybridisation in hybridisation buffer containing 47% formamide for 1 h at 37°C, the sections were hybridised overnight at 42°C with 1 ng $ml^{-1}$ ER oligoprobe. Control sections were incubated in hybridisation buffer containing 0.75 ng $ml^{-1}$ thyroglobulin probe. Additional control sections were digested by RNAse A 1 mg $ml^{-1}$ for 1 h at 37°C prior to hybridisation, and others were hybridised in the absence of labelled probe.

After hybridisation, sections were washed in graded concentrations of standard sodium citrate and incubated with an alkaline phosphatase linked antidigoxigenin antibody (Boehringer Mannheim). The reaction was developed by overnight incubation with 0.16 mg $ml^{-1}$ bromochloro-indoylphosphate and 0.33 mg $ml^{-1}$ nitroblue tetrazolium.

### *Tumours studied*

Nine breast carcinomas were selected to represent a range of ER status as measured by enzyme immunosorbent assay (EIA). This group included three breast carcinomas with no detectable ER, three with high (>300 fmol mg cytosol liquidised tissue) and three with intermediate (43–100 fmol $mg^{-1}$) ER levels.

We used archival material from 20 adenocarcinomas of the cervix from patients for whom follow-up was available. The tumours were selected to represent various morphological subtypes and grades of differentiation, and comprised 15 tumours of endocervical type, 3 clear cell carcinomas, 1 endometrioid carcinoma and 1 tumour of mixed endocervical/intestinal differentiation.

### Results

Positive staining for ER protein was denoted by brown intranuclear staining while ER negative nuclei stained pale green. ER mRNA was demonstrable as dark blue cytoplasmic staining. The sections were evaluated without prior

Correspondence: S.M. Ismail, Department of Pathology, University of Wales College of Medicine, Heath Park, Cardiff CF4 4XN, UK.
Received 19 March 1992; and in revised form 4 June 1992.

knowledge of the biochemical status of these tumours and the presence or absence of staining assessed subjectively. The heterogeneity or otherwise of staining pattern was also noted.

Immunocytochemical staining was abolished when ER antibody was replaced by control antibody. Cytoplasmic mRNA staining was greatly diminished or abolished by preincubation with RNAse. No staining was seen when the ER probe was replaced by the control thyroglobulin probe or when the test probe was omitted.

### *Breast carcinomas*

All three breast carcinomas with a high ER status by EIA showed strong positive staining for ER protein in the majority of tumour nuclei and strong cytoplasmic staining for ER mRNA (Figure 1). In those breast carcinomas with an intermediate biochemical ER status, both immunocytochemistry and *in situ* hybridisation showed more variable results with a high proportion of cells showing weak staining. In the three tumours with biochemically undetectable ER, immunocytochemistry showed large areas of negative staining with a few positive foci; the mRNA staining was indistinguishable from the intermediate group.

### *Cervical adenocarcinomas*

Six of 20 cervical adenocarcinomas were ER positive on immunocytochemistry (Figure 2); in five tumours ER protein was demonstrated in the majority of epithelial nuclei throughout the tumour, while one tumour showed focal staining. There was no relationship between ER immunoreactivity and morphological subtype of tumour; all ER positive tumours were well or moderately differentiated. Cervical stromal fibroblasts and smooth muscle cells were consistently strongly ER positive. Normal endocervical epithelial cells showed variable ER positivity.

All the cervical adenocarcinomas were positive for ER mRNA (Figure 3) while morphologically normal endocervical epithelium was negative (Figure 4). Four cervical adenocarcinomas showed an heterogeneous staining pattern with reduction of mRNA staining in poorly differentiated areas. Cervical stromal fibroblasts and smooth muscle cells showed consistent cytoplasmic positivity.

Table I summarizes the age of the patients and clinical outcome according to ER status. Fourteen patients survived disease free for a mean of 51 months following presentation (range 5–144), while five patients died of the disease (mean 11.6 months; range 2–28 months) and another died of treatment complications. All six patients whose tumours contained immunocytochemically demonstrable ER protein remained disease free on follow-up for a mean of 43.5 months. Three of four patients with cervical adenocarcinomas showing a heterogeneous staining pattern for ER mRNA died of the disease within a mean of 9 months (range 2–15) of presentation, the fourth was alive and disease free at 14 months. In contrast, of 16 patients with tumours showing diffuse ER mRNA staining, only two died of the disease.

### Discussion

Oestrogen receptor status has been found to be a useful indicator of prognosis and response to endocrine manipulation in breast carcinoma. Like the breast, the uterine cervix is a target tissue for steroid hormones and contains oestrogen receptors in endocervical epithelium, stromal cells and basal layers of squamous epithelium (Kupryjanczyk & Möller, 1988; Cano *et al.*, 1990; Nonogaki *et al.*, 1990). In this preliminary study we have shown, as far as we are aware for the first time, that a proportion of cervical adenocarcinomas also contain immunocytochemically detectable ER protein; this finding was associated with a good prognosis. We have further demonstrated that cervical adenocarcinomas contain ER mRNA a heterogeneous staining pattern for which was associated with a poor prognosis.

Previous studies using the dextran coated charcoal method (Martin *et al.*, 1978; Ford *et al.*, 1983; Gao *et al.*, 1983;

**Figure 1** A breast carcinoma with high ER levels by enzyme immunosorbent assay showing: **a**, strong widespread immunocytochemical staining for ER protein within nuclei of tumour cells. **b**, strong cytoplasmic staining for ER mRNA. × 200.

**Figure 2** Adenocarcinoma of the cervix showing intranuclear localisation for ER protein. Cervical stromal fibroblasts and smooth muscle showed consistent positive staining. × 200.

**Figure 3** Same tumour as in Figure 2 showing cytoplasmic staining for ER mRNA. × 40.

**Figure 4** Cervical adenocarcinoma showing cytoplasmic staining for ER mRNA while the adjacent normal epithelium is negative. × 180.

Martin *et al.*, 1986; Potish *et al.*, 1986; Hunter *et al.*, 1987; Harding *et al.*, 1990) have reported a variable incidence of ER positivity in cervical adenocarcinoma ranging from 23% to 81%. These figures are difficult to interpret because of variations among authors in the precise definition of receptor positivity, the sampling problems inherent in biochemical techniques which are only applicable to homogenised tissues and the ER content of the normal cervix. The immunocytochemical method we have used allows assessment of the location of ER protein, whether in tumour or in normal tissue, and has the additional advantage of being applicable to archival material. In the only study which uses a comparable method (Nonogaki *et al.*, 1990) none of the six cervical adenocarcinomas examined showed ER immunoreactivity.

All 20 tumours in our study showed cytoplasmic mRNA staining while only six contained immunocytochemically detectable ER protein. Thus there was an imbalance between ER mRNA and protein detection in cervical adenocarcinomas. Comparable findings have been reported for ER protein and mRNA in breast carcinomas (Graham *et al.*, 1991), and calcitonin peptide and mRNA in medullary carcinomas of the thyroid (Boultwood *et al.*, 1990). In contrast to the cervical adenocarcinomas, we found no demonstrable ER mRNA in normal endocervical epithelium which nevertheless did contain immunoreactive ER protein. Similarly, little or no calcitonin mRNA staining was found in normal thyroid C cells which contain abundant calcitonin peptide (Boultwood *et al.*, 1990). The paucity of mRNA in normal tissues may be explained by the relatively low turnover of protein or peptide under physiological conditions while the relative excess of mRNA in neoplasms may be caused by increased stability of mRNA in tumours, increased protein breakdown, or structural abnormalities in mRNA which interfere with protein production. Existing evidence provides examples of all these possibilities. For instance, increased stability of c-myc mRNA has been observed in Burkitt's lymphoma (Eick *et al.*, 1985). In the human papillomavirus (HPV) containing HeLa cervical carcinoma cell line, increased protein breakdown is thought to account for the absence of p53 protein in the presence of translatable p53 mRNA (Matlashewski *et al.*, 1986; Scheffner *et al.*, 1990). Finally, several variant forms of ER mRNA and protein have been described (Murphy, 1990; Fuqua *et al.*, 1991) which do not affect the region probed in our study,but which may lead to the production of an abnormal ER protein which cannot be detected by the standard antibody. The present study provides no insight as to which of these possible mechanisms are responsible for the disparity between ER mRNA and protein detection in cervical adenocarcinoma.

The oestrogen receptor is a complex protein the physiology and pathology of which remain to be fully elucidated. However, empirical detection of ER protein has provided an insight into the pathogenesis of breast carcinoma and useful guidance in its management. The findings of this preliminary study raise the possibility that oestrogens may play a comparable pathogenetic role in some cervical adenocarcinomas and that anti-oestrogen therapy may be useful in a proportion of these tumours.

We thank Dr R.I. Nicholson for information regarding the biochemical ER levels on our series of breast carcinomas and Dr J Gee for technical advice with ER immunocytochemistry.

**Table I** Oestrogen receptor protein content, age and clinical outcome in the cervical adenocarcinomas

| ER status | Number of patients | Mean age (years) [range] | Disease free | | Dead of disease | |
|---|---|---|---|---|---|---|
| | | | Number | Follow-up[b] | Number | Survival[b] |
| ICC positive | 6 | 48.3 [29–83] | 6 | 43.5 | 0 | – |
| ICC negative | 14[a] | 51.7 [33–74] | 8 | 56.6 | 5 | 11.6 |
| mRNA diffuse | 16[a] | 48.9 [29–83] | 13 | 53.8 | 2 | 20 |
| mRNA heterogeneous | 4 | 57.8 [49–74] | 1 | 14 | 3 | 9 |
| Total | 20[a] | 50.7 [29–83] | 14 | 51 | 5 | 11.6 |

[a]These figures include one patient who died of treatment complications and who therefore does not appear in the survival data. [b]Duration in months.

### References

ANDERSEN, J., ØRNTOFT, T. & POULSEN, H.S. (1986). Semiquantitative oestrogen receptor assay in formalin-fixed paraffin sections of human breast cancer tissue using monoclonal antibodies. *Br. J. Cancer*, **53**, 691–694.

BOULTWOOD, J., WYFORD-THOMAS, D., RICHARDS, G.P., CRAIG, R.K. & WILLIAMS, E.D. (1990). *In-situ* analysis of calcitonin and CGRP expression in medullary thyroid carcinoma. *Clin. Endocrinol.*, **33**, 381–390.

CANO, A., SERRA, V., RIVERA, J., MONMENEU, R. & MARZO, C. (1990). Expression of estrogen receptors, progesterone receptors, and an estrogen receptor-associated protein in the human cervix during the menstrual cycle and menopause. *Fertil. Steril.*, **54**, 1058-1064.

CHENG, L., BINDER, S.W., FU, Y.S. & LEWIN, K.J (1988). Demonstration of estrogen receptors by monoclonal antibody in formalin-fixed breast tumors. *Lab. Invest.*, **58**, 346-353.

CHILVERS, C., MANT, D. & PIKE, M.C. (1987). Cervical adenocarcinoma and oral contraceptives. *Br. Med. J.*, **295**, 1446-1447.

EICK, D., PIECHACZYK, M., HENGLEIN, B. & 6 others (1985). Aberrant c-myc RNAs of Burkitt's lymphoma cells have longer half-lives. *EMBO J.*, **4**, 3717-3725.

FARQUHARSON, M., HARVIE, R. & MCNICOL, A.M. (1990). Detection of messenger RNA using a digoxigenin end labelled oligodeoxynucleotide probe. *J. Clin. Pathol.*, **43**, 424-428.

FORD, L.C., BEREK, J.S., LAGASSE, L.D., HACKER, N.F., HEINS, Y.L. & DELANGE, R.J. (1983). Estrogen and progesterone receptor sites in malignancies of the uterine cervix, vagina, and vulva. *Gynecol. Oncol.*, **15**, 27-31.

FUQUA, S.A.W., FITZGERALD, S.D., CHAMNESS, G.C. & 5 others (1991). Variant human breast tumor estrogen receptor with constitutive transcriptional activity. *Cancer Res.*, **51**, 105-109.

GAO, Y.L., TWIGGS, L.B., LEUNG, B.S. & 5 others (1983). Cytoplasmic estrogen and progesterone receptors in primary cervical carcinoma: Clinical and histopathologic correlates. *Am. J. Obstet. Gynecol.*, **146**, 299-306.

GRAHAM, D.M., JIN, L. & LLOYD, R.V. (1991). Detection of estrogen receptor in paraffin-embedded sections of breast carcinoma by immunocytochemistry and *in situ* hybridization. *Am. J. Surg. Pathol.*, **15**, 475-485.

GREEN, S., WALTER, P., KUMAR, V. & 4 others (1986). Human oestrogen receptor cDNA: sequence, expression and homology to v-erb-A. *Nature*, **320**, 134-139.

HARDING, M., MCINTOSH, J., PAUL, J. & 5 others (1990). Oestrogen and progesterone receptors in carcinoma of the cervix. *Clin. Oncol.*, **2**, 313-317.

HUNTER, R.E., LONGCOPE, C. & KEOUGH, P. (1987). Steroid hormone receptors in carcinoma of the cervix. *Cancer*, **60**, 392-396.

KUPRYJANCZYK, J. & MOLLER, P. (1988). Estrogen receptor distribution in the normal and pathologically changed human cervix uteri: An immunohistochemical study with use of monoclonal anti-ER antibody. *Int. J. Gynecol. Pathol.*, **7**, 75-85.

MARTIN, J.D. & HÄHNEL, R. (1978). Oestrogen receptor studies in carcinoma of the endometrium, carcinoma of the uterine cervix and other gynaecological malignancies. *Aust. N.Z. J. Obstet. Gynaecol.*, **18**, 55-59.

MARTIN, J.D., HAHNEL, R., MCCARTNEY, A.J. & DE KLERK, N. (1986). The influence of estrogen and progesterone receptors on survival in patients with carcinoma of the uterine cervix. *Gynecol. Oncol.*, **23**, 329-335.

MATLASHEWSKI, G., BANKS, L., PIM, D. & CRAWFORD, L. (1986). Analysis of human p53 proteins and mRNA levels in normal and transformed cells. *Eur. J. Biochem.*, **154**, 665-672.

MURPHY, L.C. (1990). Estrogen receptor variants in human breast cancer. *Mol. Cell. Endocrinol.*, **74**, C83-C86.

NONOGAKI, H., FUJII, S., KONISHI, I. & 4 others. (1990). Estrogen receptor localization in normal and neoplastic epithelium of the uterine cervix. *Cancer*, **66**, 2620-2627.

PELLETIER, G., LIAO, N., FOLLEA, N. & GOVINDAN, M.V. (1988). Distribution of estrogen receptors in the rat pituitary as studied by *in situ* hybridization. *Mol. Cell. Endocrinol.*, **56**, 29-33.

PETERS, R.K., CHAO, A., MACK, T.M., THOMAS, D., BERNSTEIN, L. & HENDERSON, B.E. (1986). Increased frequency of adenocarcinoma of the uterine cervix in young women in Los Angeles County. *J. Natl. Cancer Inst.*, **76**, 423-428.

PONGLIKITMONGKOL, M., GREEN, S. & CHAMBON, P. (1988). Genomic organization of the human oestrogen receptor gene. *EMBO J.*, **7**, 3385-3388.

POTISH, R.A., TWIGGS, L.B., ADCOCK, L.L., PREM, K.A., SAVAGE, J.E. & LEUNG, B.S. (1986). Prognostic importance of progesterone and estrogen receptors in cancer of the uterine cervix. *Cancer*, **58**, 1709-1713.

SCHEFFNER, M., WERNESS, B.A., HUIBREGTSE, J.M., LEVINE, A.J. & HOWLEY, P.M. (1990). The E6 oncoprotein encoded by human papillomavirus types 16 and 18 promotes the degradation of p53. *Cell*, **63**, 1129-1136.

SCHWARTZ, S.M. & WEISS, N.S. (1986). Increased incidence of adenocarcinoma of the cervix in young women in the United States. *Am. J Epidemiol.*, **124**, 1045-1047.

WAKEFIELD, E.A. & WELLS, M. (1985). Histochemical study of endocervical glycoproteins throughout the normal menstrual cycle and adjacent to cervical intraepithelial neoplasia. *Int. J. Gynecol. Pathol.*, **4**, 230-239.